# Comparison between intrathecal morphine and intravenous patient control analgesia for pain control after video-assisted thoracoscopic surgery: A pilot randomized controlled study

**DOI:** 10.1371/journal.pone.0266324

**Published:** 2022-04-06

**Authors:** Amorn Vijitpavan, Nussara Kittikunakorn, Rojnarin Komonhirun

**Affiliations:** Department of Anesthesiology, Faculty of Medicine Ramathibodi Hospital, Mahidol University, Bangkok, Thailand; Ziekenhuis Rijnstate Arnhem, NETHERLANDS

## Abstract

**Background:**

Video-assisted thoracoscopic surgery (VATS) is a minimally invasive procedure, but patients may still experience intense pain, especially during the early postoperative period. Intrathecal morphine (ITM) is an effective pain control method that involves a simple maneuver and has a low risk of complications. This study aimed to study the effectiveness of ITM for pain control in patients who undergo VATS.

**Materials and methods:**

A randomized controlled study was conducted who were in ASA classes 1–3, aged over 18 years, and scheduled for elective VATS. Patients were randomized into two groups: the ITM group (n = 19) received a single shot of 0.2 mg ITM before general anesthesia; and the control group (n = 19) received general anesthesia only. For 48 hours after surgery, other than intravenous patient-controlled analgesia (IVPCA) morphine, patients received no sedatives or opioid medications except for 500 mg acetaminophen four times daily orally. Postoperative pain scores and IVPCA morphine used, side effects, sedation at specific time-points, i.e., 1, 6, 12, 24, and 48-hours and overall treatment satisfaction scores were assessed.

**Results:**

Postoperative pain scores (median [IQR]) in ITM group were significantly lower than control group (repeated-measure ANOVA, *p* = 0.006) and differed at the first (7 [2, 7] vs 8 [6, 9], *p* = 0.007) and sixth hours (3 [2, 5] vs 5 [5, 7], *p* = 0.002). The cumulative dose of post-operative morphine (median [IQR]) in ITM group was also lower (6 [3, 20] vs 19 [14, 28], *p* = 0.006). The incidence of pruritus was significantly higher in ITM group (68.42% vs. 26.32%, *p* = 0.009). No significant differences in nausea and vomiting, sedation scores, and satisfaction scores were observed between the two groups.

**Conclusion:**

ITM could reduce pain scores and opioid consumption after VATS compared to IVPCA-opioids. However, pain scores and opioid consumption still remained high. No difference in patient satisfaction was detected.

## Introduction

Video-assisted thoracoscopic surgery (VATS), which is considered a minimally invasive approach, is widely performed in place of procedures that formerly required open thoracotomy [1, 2]. Its key advantages over other traditional approaches include: (1) less postoperative pain; (2) fewer pulmonary complications; (3) shorter hospital stay with reduced costs; and (4) a cosmetic incision [3, 4]. Despite such distinct benefits, some patients experience considerable pain after undergoing VATS, especially during the early postoperative period [5–7]. Numerous researchers have previously investigated the best approach for pain control following VATS [8–12]. Thoracic epidural analgesia (TEA), which is the gold standard for pain control for open thoracotomy [13], has also been applied to postoperative analgesia for VATS. However, TEA for postoperative pain control is not appropriate for minimally invasive procedures such as VATS, which involves limited tissue trauma and lower pain severity [14]. Several studies have shown that paravertebral blocks are at least as effective as TEA in patients undergoing VATS [15–17]. Fascial plane, erector spinae plane [18–20], and serratus anterior plane blockades [21, 22] have also been implemented for VATS for postoperative pain control, and they have been recommended as part of a multimodal approach for postoperative analgesia. However, each institution continues to use different methods of pain relief following VATS [21–23]. Intrathecal morphine (ITM) is not only simple but also has a low risk of technical complications or failure. It is a cost-effective method that is widely used for pain control for many procedures [24–26]. Moreover, it provides analgesia without inducing motor and sensory deficits [27]. It is a single shot technique which has several benefits over a catheter technique with respect to mobilization. The approach of ITM combined with a local anesthetic agent for postoperative analgesia has been widely used for pain control for numerous operations. However, ITM without a local anesthetic is also used as a single-dose injection with general anesthesia to prevent pain following major surgery [27–30]. Therefore, ITM may be an effective pain control regimen for patients who undergo VATS and require early ambulation. This research aimed to study the efficacy of ITM as postoperative analgesia for VATS.

## Materials and methods

### Study design and participants

This study was a pilot prospective randomized controlled study conducted in Thai patients scheduled for elective VATS at Ramathibodi Hospital. The study obtained approval from the Committee on Human Rights Related to Research Involving Human Subjects, Faculty of Medicine, Ramathibodi Hospital (approval certificate ID: MURA2015/403; protocol ID 07-58-07; approved July 15, 2015). The first subject was recruited on August 17, 2015, and the final subject was followed up on May 12, 2016. The study was registered on the Thai Clinical Trial Registry (TCTR) at http://www.clinicaltrials.in.th (TCTR20201005002). The study was retrospectively registered because it was not a requirement for obtaining study approval and miscommunication between study team. The authors confirm that all ongoing and related trials for this drug/intervention are registered.

Inclusion criteria were patients undergoing elective VATS, who were aged over 18 years, and had an American Society of Anesthesiologists (ASA) physical status I to III. Exclusion criteria were unwillingness and/or inability to provide written informed consent to participate in the study, pregnancy, contraindication to spinal block, history of allergy to opioids, receiving opioid therapy for chronic pain, history of severe postoperative nausea and vomiting (PONV) that required treatment, history of previous surgical intervention at spinal T12–L5 level or significant spinal deformity, learning difficulties or communication problems, and retaining the endotracheal tube after the operation.

### Randomization, blinding, and interventions

The eligible patients were randomized using the sealed envelope technique on the day of VATS into either Group 1 (ITM group), who received 0.2 mg ITM, or Group 2 (control group), who received intravenous (IV) patient control analgesia (IVPCA) morphine, at an allocation ratio of 1:1. The randomization scheme was generated by an independent person involved in the study (departmental secretary) using the website, Randomization.com (http://www.randomization.com) with a block size of two. Assignments were made based on sequentially numbered randomization numbers on the randomization envelopes. Randomization envelopes were prepared and sealed by the independent person involved in the study. The study was a single-blind study. There was an inability to blind patients, surgeons, anesthesiologists, and inpatient care nurses/staff in the ITM group from administering ITM and the lack of intrathecal placebo in the control group. Only the outcome assessor, an acute pain nurse, was blinded to the group assignment.

### Anesthesia management

All patients received 0.05 mg/kg IV midazolam for sedation in the holding area 15 minutes before being transferred to the operating room. In the ITM group, the intrathecal administration of morphine was performed before the induction of general anesthesia, with patients lying in a lateral position. Ten mg/ml of morphine was prepared as 1 mg/ml, with 9 ml of 0.9% sodium chloride in a 10-ml syringe. Then, 1 ml of this concentration was aspirated and diluted with 0.9% sodium chloride to provide 10 ml [31]. After mixing, 2 ml (0.2 mg) of this mixture was aspirated with a 5-ml syringe and injected into the intrathecal space at L3–4 or the L4–5 interspace, using a 27 G spinal needle (Spinocan 27G × 4 ¾ in). The control group did not receive ITM. In the event of a traumatic or bloody tap, surgery was postponed. The intraarterial line was placed in patients of both groups to monitor hemodynamics. After pre-oxygenation, anesthetic induction was performed with a bolus injection of propofol (1–2 mg/kg) or thiopental (3–5 mg/kg), fentanyl (2 μg/kg), and atracurium (0.6 mg/kg) or cisatracurium (0.15 mg/kg). Cisatracurium or atracurium were administered every 30 minutes. Maintenance of anesthesia was achieved with 1%–2% sevoflurane or 5%–6% desflurane according to surgical stimuli and hemodynamic response, and a muscle relaxant was given as needed. No other narcotics except for fentanyl were used during surgery. Both groups received 2 ml/kg/hour of acetate Ringer’s solution for intraoperative period fluid maintenance, and the amount was increased according to blood loss. At the end of the procedure, 0.02 mg/kg atropine and 0.05 mg/kg neostigmine were given to reverse the muscle relaxant. Patients were awakened and extubated if standard extubation criteria were met.

### Surgical management

After induction and intubation, urinary catheters were inserted into the patients, who were then positioned into a lateral position. Two experienced thoracic surgeons, who were not involved in the study, performed VATS using the two thoracoscopic portal methods. The camera port was incised at a width of 1 cm at the sixth intercostal space anterior axillary line. The port for the surgical instrument was opened to a width of 4 cm at the fourth intercostal space anterior axillary line, and a rib spreader was not used. After completing the operation, a chest tube was placed in the camera port and removed on postoperative days 2–4, depending on postoperative bleeding and air leakage. No local anesthetic agent infiltrated the portal wounds before the incision or after completion of the procedure.

### Postoperative management

Patients were then transferred to a recovery room for close monitoring for 1 hour before being moved to the ward, in accordance with hospital protocol. All patients received an oxygen mask with a nebulizer flow of 8–10 L/min and fraction of inspired oxygen of 0.4 to maintain blood oxygen saturation (SpO_2_) at over 90%. IVPCA was then started using a patient-controlled anesthesia (PCA) device, programmed to deliver morphine at a concentration of 1 mg/ml, 1 mg/dose, and no basal rate, with lockout duration of 5 minutes. The limit for 4 hours was 40 mg. Other than IVPCA morphine, patients received neither sedatives nor opioids. During the postoperative period, all patients were given 500 mg acetaminophen four times daily orally for 48 hours. Oxygenation, ventilation and level of consciousness were monitored once every hour for the first 12 hours, once every 2 hours for the next 12 hours and once every 4 hours for the next 24 hours until 48 hours. All patients were encouraged by physical therapists to ambulate, as per the thoracic surgery protocol of the hospital.

The side effect of pruritus was treated if the patient experienced symptoms and requested medication. Pruritus was treated with 10 mg of IV chlorpheniramine. PONV was treated with 4 mg IV ondansetron if the patient had nausea symptoms and requested treatment or experienced vomiting.

Sedation was monitored and assessed by a nurse using Ramsay’s score. If the patient’s sedation score was > 2 or SpO_2_ was < 94%, an oxygen mask was provided to the patient, and the on-call doctor was notified. Respiratory depression was evaluated by clinical observation by a nurse, and the on-call doctor was notified if the respiratory rate became lower than eight breaths/minute. The urinary catheter was retained for 24 hours, as per the hospital protocol for thoracotomy.

### Outcome measurements

Primary outcome was pain at a specific time point, i.e. 1, 6, 12, 24, and 48 hours post-operation. Secondary outcomes were IVPCA morphine consumption and incidence of common side effects of opioid analgesia, i.e. pruritis, nausea/vomiting and sedation. The outcomes were evaluated by an acute pain nurse who was not involved in the study drug allocation, administration, inpatient caring and had no knowledge of the patient groupings.

An 11‐point numeric pain rating scale (NRS) from 0 = no pain at all to 10 = worst possible pain imaginable, was used to measure pain intensity at the specific time-points. IVPCA morphine consumption and incidence of common side effects (PONV and pruritus) were also recorded at the same time points.

PONV and pruritus were recorded simultaneously using a three-point ordinal scale (0 = no symptoms, 1 = symptoms without treatment, and 2 = symptoms with treatment). Patient sedation was evaluated using the five-point sedation Ramsay’s score [32] (1 = wide awake, 2 = drowsy or dozing intermittently, 3 = mostly sleeping but easily awakened, 4 = asleep, difficulty responding to verbal commands, and 5 = awakened only by shaking). In cases of excessive sedation (sedation score of ≥ 4), alongside a respiratory rate of ≤ eight breaths/min, IV naloxone was administered. The total amount of morphine given and satisfaction, rated on a five-point patient satisfaction scale (1 = very dissatisfied, 2 = dissatisfied, 3 = neutral, 4 = satisfied, and 5 = very satisfied), at 48 hours post-VATS were recorded.

### Statistical analysis

In a previous study [33], means (± standard deviations, SD) of postoperative pain intensity at rest evaluated using a visual analog scale (VAS) from 0 to 10 on day 1 were 2.4 ± 1.39 in the ITM group and 4.1 ± 2.19 in the general anesthesia group. Therefore, we expected the difference in mean pain intensity to be 1.7 and the within-group standard deviation to be 1.8 ([1.39 + 2.19]/2). Power and Sample Size Calculation Program version 3.1.2 was used to estimate the sample size for a 1:1 ratio of control and experimental patients, which showed that 19 subjects would be needed per group to reject the null hypothesis that the means of the experimental and control groups are equal, with a power of 80% and a type-I error probability of 0.05.

All statistical analyses were carried out using STATA 15.1 (StataCorp, College Station, TX, USA). Descriptive statistic, i.e., frequency, means (±SD), median with interquartile ranges (IQR), and/or minimum and maximum (min-max) were used to described the data. Continuous data were compared using the Mann-Whitney test. Comparisons of categorical data were performed using chi-square or Fisher’s exact tests, as appropriate. Interval data (pain score and morphine consumption across time) were analyzed for between-group effects using a repeated-measures analysis of variance (repeated- measure ANOVA). Probability (*p*) values < 0.05 were considered significant.

## Results

### Participants, baseline demographics, and characteristics

**Fig 1:**
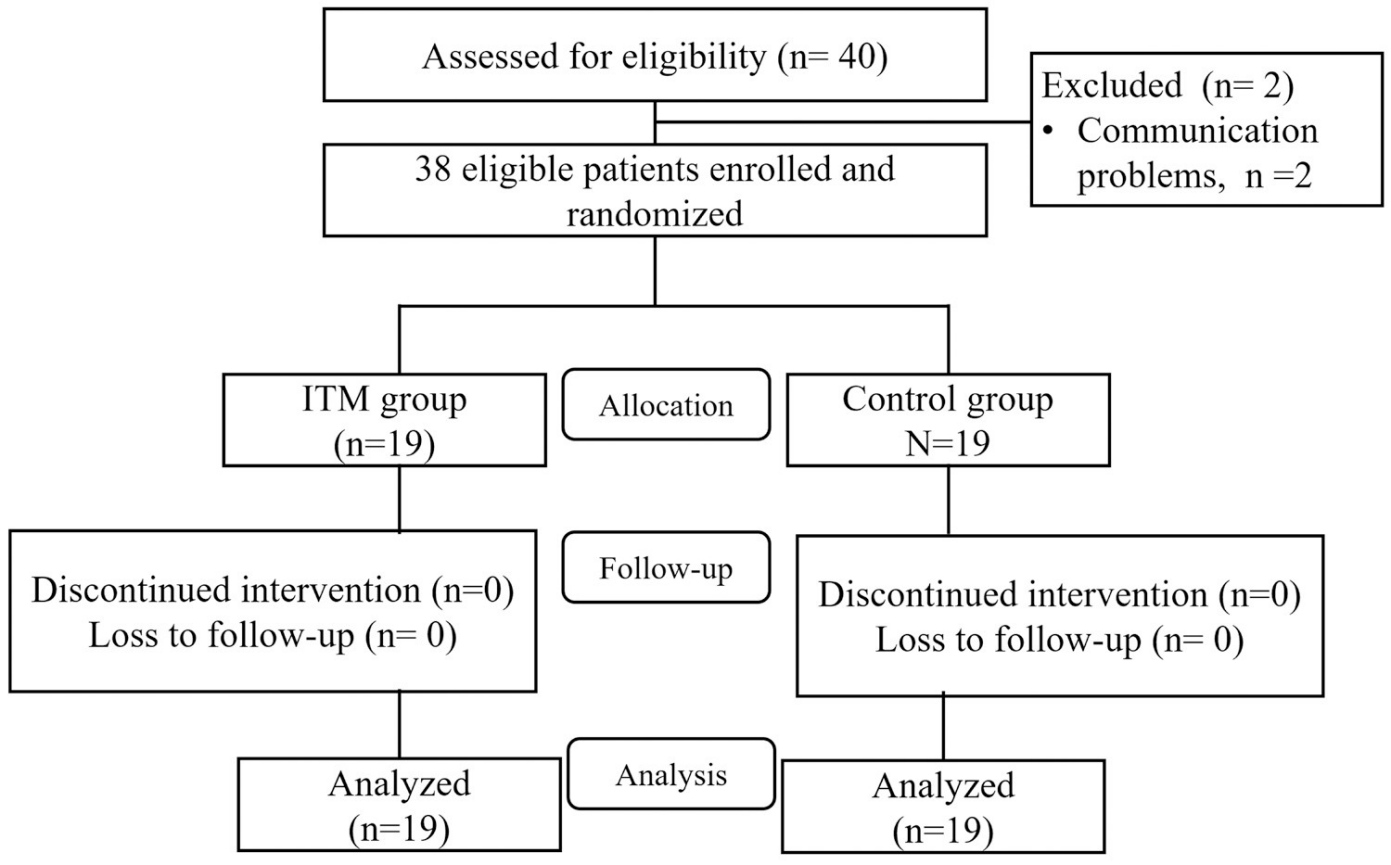
Participant flowchart.

Between March 2015 and June 2016, 38 patients were randomized into either the ITM or control group (19 patients per group). None of the patients dropped out of the study (Fig 1). The demographic data by groups, i.e., sex, age, body mass index [BMI], ASA physical status, underlying disease, smoking, blood loss, fluid replacement, and vasopressor used are presented in Table 1. None of the patients required an extended observation period in PACU, and none required blood transfusions, reoperation, or readmission. Both groups had similar types of operations and were not significantly different in terms of duration of surgery or hospital stay.

**Table 1:** Pre-operative demographics and characteristics.

Variables	ITM group (n = 19)	Control group (n = 19)
Sex, n (%)		
Male	10 (52.63%)	13 (68.42%)
Female	9 (47.37%)	6 (31.58%)
Age (years), median (min–max)	64 (22–70)	57 (18–69)
BMI (kg/m^2^), median (min–max)	22.8 (16.3–33)	21.3 (17.3–31.3)
Laboratory test		
Hb (g/dl), median (min–max)	13 (10.2–16)	13.3 (9–15)
WBC (cells/mm^3^), median (min–max)	6750 (4430–8700)	6550 (4500–8960)
Platelet (cells/mm^3^) median (min–max)	279000 (187400–454000)	335000 (200000–396000)
Albumin (unit), median (min–max)	3.5 (2.6–4.2)	3.8 (3–4.6)
ASA physical status, n (%)		
I	2 (10.53%)	4 (21.05%)
II	10 (52.63%)	9 (47.37%)
III	7 (36.84%)	6 (31.58%)
Underlying disease(s), n (%)		
Diabetes mellitus	4 (21.05%)	1 (5.26%)
Hypertension	8 (42.11%)	5 (26.32%)
Dyslipidemia	8 (42.11%)	3 (15.79%)
Smoking, n (%)	4 (21.05%)	8 (42.11%)
Vasopressor, n (%)	5 (26.32%)	4 (21.05%)
Fluid (ml), median (min–max)	770 (440–1550)	770 (500–1600)
Chest drain, n (%)		
2 days	10 (52.63%)	8 (42.11%)
3 days	7 (36.84%)	7 (36.84%)
4 days	2 (10.53%)	4 (21.05%)
Operation, n (%)		
Wedge resection	4 (21.05%)	3 (15.79%)
Segmentectomy	2 (10.53%)	2 (10.53%)
Lobectomy	6 (31.58%)	7 (36.84%)
Pleurectomy	3 (15.79%)	4 (21.05%)
Thymectomy	4 (21.05%)	3 (15.79%)
Tumor	12 (63.16%)	12 (63.16%)
Blood loss (ml), median (min–max)	150 (50–300)	100 (50–400)
Blood transfusion, n (%)	0 (0%)	0 (0%)
Surgical duration (min), median (min–max)	178 (90–230)	175 (100–250)
Reoperation, n (%)	0 (0%)	0 (0%)
Hospital stay (days), median (min–max)	4 (3–5)	4 (3–6)

**Abbreviations:** ASA, American Society of Anesthesiologists; BMI, body mass index; Hb, hemoglobin

WBC, white blood cell; ITM, intrathecal morphine

### Outcomes

#### Pain scores and IVPCA morphine

Overall postoperative pain scores and amount of IVPCA morphine of the ITM group were significantly lower than those of the control group (repeated-measure ANOVA, *p* = 0.006 and *p* = 0.029, respectively). The pain scores differed significantly 1 hour after VATS (median [IQR] (min-max): 7 [2, 7] (0–9) vs 8 [6, 9] (5–10), *p* = 0.007) and 6 hours (median [IQR]: 3 [2, 5] (0–7) vs 5 [5, 7] (2–9), *p* = 0.002). However, there were no differences in pain scores 12-, 24-, or 48-hours post-operation. The demand for morphine was significantly lower in the ITM group than in the control group at all measurement points on the first postoperative day. However, after 24 hours, morphine demand did not significantly differ between groups (Table 2).

**Table 2:** Post-operative outcomes (pain, sedation, and satisfaction scores).

Variables	ITM group (n = 19)	Control group (n = 19)	*P*-value^a^	*P*-value^b^
Post-operative pain score, median (min–max)				0.006*
At 1 hour	7 (0–9)	8 (5–10)	0.007*	
At 6 hours	3 (0–7)	5 (2–9)	0.002*	
At 12 hours	3 (0–8)	4 (1–8)	0.148	
At 24 hours	3 (0–7)	4 (1–8)	0.561	
At 48 hours	2 (0–6)	3 (0–7)	0.075	
Amount of IVPCA morphine (mg), median (min–max) for each assessment period				0.029*
0 to 1st hour	3 (0–18)	8 (1–20)	0.034*	
From the 1st–6th hours	0 (0–12)	2 (0–6)	0.024*	
From the 6th–12th hours	1 (0–9)	2 (0–10)	0.023*	
From the 12th–24th hours	1 (0–7)	2 (0–11)	0.006*	
From the 24th–48th hours	0 (0–24)	6 (0–31)	0.103	
Cumulative amount of IVPCA morphine used in 48 hours (mg), median (min–max)	6 (0–54)	19 (4–63)	0.006*	
Time to first morphine administration after the operation (hours), median (min–max)	1 (0–14)	1 (1–4)	0.829	
Overall satisfaction score, median (min–max)	5 (3–5)	4 (1–5)	0.653	

^a^ Mann-Whitney tests to compare differences of the data between two groups.

^b^ Repeated measures analysis of variance testing effect of intrathecal morphine (ITM) on overall Hb level.

**Abbreviations**: IVPCA, intravenous patient-controlled analgesia; ITM, intrathecal morphine

*p < 0.05.

The cumulative dose of morphine used within the first 48 hours post-operation in the ITM group was significantly lower than that in the control group (median [IQR] (min-max): 6 [3, 20] (0, 54) vs. 19 [14, 28] (4, 63), *p* = 0.006). No difference in the time to first postoperative morphine administration was observed between the groups. The subgroup comparison of pain scores between tumor surgery (lobectomy, segmentectomy, and wedge resection) and pleurectomy showed no significant differences.

### Common side effects

The overall incidence of pruritus 48 hours after VATS in the ITM group was significantly higher than that in the control group (13/19 [68.42%] vs. 5/19 [26.32%], *p* = 0.009). However, there were no significant differences in the incidence of sedative effects or PONV. The incidence of pruritus was significantly higher in the ITM group than in the control group 6 hours (9/19 [47.37%] vs. 5/19 [5.26%], *p* = 0.003) and 12 hours (12/19 [63.16%] vs. 0/19 [0%], *p* < 0.001) post-operation (Table 3). None of the patients in either group had respiratory depression.

**Table 3:** Common opioid-related side effects (sedative effect, nausea/vomiting, and pruritus).

Variables	ITM group (n = 19)	Control group (n = 19)	*P*-value^a^
**Sedative effect**			
Having sedative event within 48 hrs, n (%)	17 (89.47)	19 (100)	0.146
Sedation, number of each score ^b^ (1/2)			
At 1 hr	3/16	0/19	0.230
At 6 hrs	14/5	14/5	1.000
At 12 hrs	18/1	17/2	1.000
At 24 hrs	19/0	19/0	-
At 48 hrs	18/1	19/0	1.000
**Pruritus**			
Having pruritus within 48 hrs post-operation, n (%)	13 (68.42)	5 (26.32)	
No symptom	6 (31.58)	14 (73.68)	
Having symptom without treatment	4 (21.05)	2 (10.53)	
Having symptom with treatment	9 (47.37)	3 (15.79)	
Pruritus, number of each score ^c^ (0/1/2)			
At 1 hr	18/0/1	19/0/0	1.000
At 6 hrs	10/6/3	18/0/1	0.005*
At 12 hrs	7/7/5	19/0/0	<0.001*
At 24 hrs	12/4/3	17/2/0	0.102
At 48 hrs	19/0/0	17/0/2	0.486
**Nausea/Vomiting**			
Having nausea/vomiting within 48 hrs post-operation, n (%)	13 (68.42)	14 (73.68)	0.721
No symptom	6 (31.58)	5 (26.32)	1.000
Having symptom without treatment	2 (10.53)	3 (15.79)	
Having symptom with treatment	11 (57.89)	11 (57.89)	
Nausea/Vomiting, number of each score^c^ (0/1/2)			
At 1 hr	17/2/0	18/0/1	0.468
At 6 hrs	10/3/6	11/5/3	0.468
At 12 hrs	9/3/7	10/3/6	1.000
At 24 hrs	12/5/2	7/7/5	0.220
At 48 hrs	17/2/0	12/4/3	0.102

^a^ Chi-squared test or Fisher’s exact tests

^b^ Sedation scores from 5-point sedation Ramsay’s score: 1 = wide awake, 2 = drowsy or dozing intermittently, 3 = mostly sleeping but easily awakened, 4 = asleep, difficulty responding to verbal commands, 5 = awakened only by shaking

^c^ Pruritus scores and Nausea/Vomiting scores; 0 = no symptom, 1 = having symptom without treatment, and 2 = having symptom with a treatment

* Significant at *P*-Value <0.05

## Discussion

The study reported no differences between the two groups for factors that may impact pain scores and morphine requirements (e.g., sex, age, BMI, chest tube, and smoking) [6, 34–38]. The pain scores of the ITM group were significantly lower than those of the IVPCA morphine group (*p* = 0.006) for two time points only, i.e., 1- and 6-hours post-operation. However, although scores were significantly lower in the ITM group than in the control group, pain scores remained high during the first hour following the operation. In the present study, we used 0.2 mg of ITM for post-VATS pain control, based on the study of Suksompong et al., which demonstrated that 0.2 mg of ITM was effective as same as 0.3 mg of ITM for postoperative pain control for standard thoracotomy with a similar degree of side effects [39]. This may be because ITM had not reached its full effect or a single dose of 0.2 mg ITM without supplementing with other analgesics was insufficient to control pain during the period immediately following the operation. The dosage of ITM for open thoracotomy varies from 5 to 12 μg/kg (0.3–1.2 mg) [40, 41]; however, although higher dosages provide more pain relief, it is at the cost of an increased incidence of pruritus and PONV and an increased risk of respiratory depression. Pain immediately post-operation can be resolved by the surgeon administering an intercostal nerve blockade under direct vision before closing the wound or by administering one or more long-acting analgesic drugs, such as opioids, IV nonsteroidal anti-inflammatory drugs (NSAIDs), or IV acetaminophen, before completing the operation to extend analgesia to early postoperative periods. Pain scores were significantly reduced to an acceptable level at 6 and 24 hours after surgery, which is consistent with the clinical duration of action of ITM of excellent analgesia for up to 18–24 hours after administration [42–44]. Although pain scores differed between groups at the first and sixth hours only, morphine consumption was significantly different between the two groups for 24 hours (*p* = 0.029). The ITM group required less morphine for postoperative pain than did the control group for up to 24 hours; however, after 24 hours, both groups had similar morphine demands. These results are consistent with previous studies on ITM use in patients undergoing other minimally invasive cardiac surgeries [45, 46] and major abdominal surgeries [47, 48]. A meta-analysis by Meylan et al. and some studies found that patients who received ITM for pain control after thoracotomy required more rescue doses of morphine than those who underwent major abdominal surgery [29, 30]. Boussofara et al. demonstrated that NSAID administration reduces opioid demand by 30%–35% [49]. In this study, we did not add NSAIDs to the protocol because we were interested in determining the actual analgesic effect of ITM for post-VATS pain control. Although we found that pain scores and the morphine demand were lower in the ITM group than in the control group, supplementary IV morphine was still required for these patients, which indicated that ITM alone was insufficient to completely relieve pain following VATS. We speculated that the pain scores and amount of IV morphine administration could be reduced if NSAIDs were given in combination with oral or IV acetaminophen [49–52]. Several previous studies have reported that the multimodal approach for VATS should be considered for post-thoracotomy analgesia [53–55]. Our subgroup comparison between the pleurectomy (bullectomy with surgical pleurodesis) and tumor surgery groups (lobectomy, segmentectomy, and wedge resection) did not reveal differences in pain score or morphine consumption. However, this finding requires validation because the number of patients in each group was small.

Pruritus is the most common adverse effect of ITM, with a reported incidence of 30%–100% [44]. In the present study, within the follow-up duration of 48 hours, over half the patients in the ITM group experienced pruritus, which was significantly higher than those who received IVPCA, especially within 24 hours post-operation, which corresponds to the duration of action of ITM. Although the incidence of pruritus requiring treatment was high (47.37%), all patients with symptoms were managed by symptomatic treatment. This study used antihistamine to treat the itching. Although it was found to be ineffective in treating itching caused by intrathecal opioids [56], the sedative effects of antihistamines may be helpful by making the patients sleepy and reducing scratching [57]. Unfortunately, our hospital doesn’t have the drugs in opioid agonist-antagonist, which is more effective to treat pruritus. Nevertheless, for patients who cannot tolerate severe pruritus symptoms and ask for more medication after receiving an antihistamine, we will administer intravenous naloxone infusion to relieve this symptom. None of the patients in this study experienced severe itching that required naloxone for treatment. Both groups had a high incidence of PONV (57.89%), although incidence did not differ between groups. Patients with severe PONV achieved symptom relief with symptomatic treatment.

No patients in this study had respiratory depression, and the sedation scores were comparable across groups. Furthermore, ITM did not impact hospital stay duration or reoperation. In terms of patient satisfaction, a single dose of 0.2 mg ITM followed by IVPCA morphine provided the maximum satisfaction score for postoperative analgesia, although scores did not differ between groups. We did not identify the cause of patient dissatisfaction, whether by pain, nausea, pruritus, or other reasons. Therefore, the ITM technique is safe for VATS. Our study demonstrated that the ITM technique, a simple procedure with a low technical failure rate and low risk of serious complications, is suitable for incorporation into a multimodal analgesic scheme for postoperative pain control, especially during the first 24 hours following VATS.

In terms of patient satisfaction, a single dose of 0.2 mg ITM followed by IVPCA morphine provided the maximum satisfaction score for postoperative analgesia; although scores did not differ between groups. We did not identify the cause of patient dissatisfaction, whether by pain, nausea, pruritus, or other reasons.

No patients in the study had respiratory depression, and sedation scores were comparable across groups. Furthermore, ITM did not impact hospital stay duration or reoperation. Therefore, the ITM technique is safe for VATS. Our study demonstrated that the ITM technique, which is a simple procedure that has a low technical failure rate and a low risk of serious complications, is suitable for incorporation into a multimodal analgesic scheme for postoperative pain control, especially during the first 24 hours following VATS.

### Limitations

We did not use an intrathecal placebo in the control group because of ethical reasons. Therefore, the patients, surgeons, anesthesiologists, and inpatient care nurses/staff were not blinded, which may introduce biases toward outcomes. The dose of 200 micrograms of morphine may be imprecision because the actual amount of morphine is very small and challenging to measure in routine clinical practice. Furthermore, various types of operations may induce different levels of pain severity, although we did not find differences in pain scores between operation types. Additionally, the number of portal incisions may impact postoperative analgesia after VATS [58–60], where the single-port technique may result in lower pain compared with the multi-port approach. Our surgeries involved two portal site incisions, which may contribute to the differences in pain scores reported in other studies that used the single port technique.

In this present study, we did not perform covariate adjustment analysis due to the small sample size. We calculated the sample size based on pain scores only. A larger sample size and covariate adjustment are suggested for future study. The pain scores and the amount of IV morphine administration in this study were still high in both groups. Adding NSAIDs for future research may reduce pain scores and the amount of morphine in the patients receiving ITM. Finally, we assessed pain by asking patients to rate their overall pain. Although pain scores were lower in the ITM group, we did not assess whether this was regarding pain at rest or during movement, which is another limitation of the study. In addition, using the single-scale satisfaction measurement to evaluate the patient satisfaction may not precise as the method of multi-scale satisfaction. The study was retrospectively registered in the TCTR Database but the study was reviewed and approved by the hospital ethical committee prior to commencing the study.

## Conclusion

This study demonstrated that ITM reduced pain scores and opioid consumption in the first 24 hours after VATS compared to solely IVPCA-opioids. However, the pain scores and opioid consumption had still remained high in the ITM group and no difference in patient satisfaction was detected. These findings suggested that ITM should not be used as a sole method of analgesia but it may have an excellent opioid-sparing effect as part of multimodal analgesia management.

### What is already known on this topic?

VATS is a minimally invasive operation for various thoracic procedures. The advantages of this technique are fewer pulmonary complications, shorter hospital stays, and faster recovery compared with open thoracotomy. However, despite these distinct benefits, postoperative pain remains a significant concern. Although we have developed numerous methods for pain control following VATS, such as thoracic epidural, paravertebral, erector spinae, serratus anterior, and intercostal nerve blocks, these techniques require a high level of skill, additional training, and an ultrasound machine to perform the procedure.

### What this study adds?

ITM is a cost-effective method for pain control without the need for additional training or expensive devices, especially during the first postoperative day following VATS. Not only is it simple but it also has a low risk of technical complications or failure. Moreover, it can provide analgesia without inducing motor and sensory deficits. Therefore, ITM should be considered as a suitable option for postoperative multimodal analgesia.

## Supporting information

S1 Dataset (XLSX)
